# Piezo1 channels are mechanosensors in human fetoplacental endothelial cells

**DOI:** 10.1093/molehr/gay033

**Published:** 2018-08-02

**Authors:** L C Morley, J Shi, H J Gaunt, A J Hyman, P J Webster, C Williams, K Forbes, J J Walker, N A B Simpson, D J Beech

**Affiliations:** 1 Leeds Institute of Cardiovascular and Metabolic Medicine, LIGHT Laboratories, University of Leeds, 6 Clarendon Way, Leeds, UK; 2 Academic Department of Obstetrics and Gynaecology, Level 9 Worsley Building, School of Medicine, University of Leeds, Leeds, UK

**Keywords:** placenta, endothelium, shear stress, mechanosensor, ion channel, calcium ion, Piezo1, foetal growth restriction

## Abstract

**STUDY QUESTION:**

Does the shear stress sensing ion channel subunit Piezo1 have an important mechanotransduction role in human fetoplacental endothelium?

**SUMMARY ANSWER:**

Piezo1 is present and functionally active in human fetoplacental endothelial cells, and disruption of Piezo1 prevents the normal response to shear stress.

**WHAT IS KNOWN ALREADY:**

Shear stress is an important stimulus for maturation and function of placental vasculature but the molecular mechanisms by which the force is detected and transduced are unclear. Piezo1 channels are Ca^2+^-permeable non-selective cationic channels which are critical for shear stress sensing and maturation of murine embryonic vasculature.

**STUDY DESIGN, SAMPLES/MATERIALS, METHODS:**

We investigated the relevance of Piezo1 to placental vasculature by studying human fetoplacental endothelial cells (FpECs) from healthy pregnancies. Endothelial cells were isolated from placental cotyledons and cultured, for the study of tube formation and cell alignment to shear stress. In addition, human placental arterial endothelial cells were isolated and studied immediately by patch-clamp electrophysiology.

**MAIN RESULTS AND THE ROLE OF CHANCE:**

The synthetic Piezo1 channel agonist Yoda1 caused strong elevation of the intracellular Ca^2+^ concentration with a 50% effect occurring at about 5.4 μM. Knockdown of Piezo1 by RNA interference suppressed the Yoda1 response, consistent with it being mediated by Piezo1 channels. Alignment of cells to the direction of shear stress was also suppressed by Piezo1 knockdown without loss of cell viability. Patch-clamp recordings from freshly isolated endothelium showed shear stress-activated single channels which were characteristic of Piezo1.

**LIMITATIONS, REASONS FOR CAUTION:**

The *in vitro* nature of fetoplacental endothelial cell isolation and subsequent culture may affect FpEC characteristics and *PIEZO1* expression. In addition to Piezo1, alternative shear stress sensing mechanisms have been suggested in other systems and might also contribute in the placenta.

**WIDER IMPLICATIONS OF THE FINDINGS:**

These data suggest that Piezo1 is an important molecular determinant of blood flow sensitivity in the placenta. Establishing and manipulating the molecular mechanisms regulating shear stress sensing could lead to novel therapeutic strategies to improve blood flow in the placenta.

**LARGE-SCALE DATA:**

Not applicable.

**STUDY FUNDING/COMPETING INTEREST(S):**

LCM was funded by a Clinical Research Training Fellowship from the Medical Research Council and by the Royal College of Obstetricians and Gynaecologists, and has received support from a Wellcome Trust Institutional Strategic Support Fund. JS was supported by the Wellcome Trust and a BHF Intermediate Research Fellowship. HJG, CW, AJH and PJW were supported by PhD Studentships from BHF, BBSRC and the Leeds Teaching Hospitals Charitable Foundation respectively. All authors declare no conflict of interest.

## Introduction

Together with the maternal uteroplacental circulation, the fetoplacental vasculature is vital for placental perfusion and therefore a critical determinant of foetal growth and a successful pregnancy outcome (Kingdom *et al.*, 2000; Su, 2015). Fetoplacental endothelial cells (FpECs) are key regulators of angiogenesis and vasomotor tone, through the production of vasoactive mediators (Su, 2015).

When blood flow through the placenta is compromised, foetal growth restriction (FGR) can result (Kingdom *et al.*, 2000; Su, 2015). Affected pregnancies are associated with an increased risk of perinatal morbidity and mortality (Alfirevic *et al.*, 2015). The lack of available treatment for FGR often necessitates delivering the baby in order to prevent foetal demise, which may result in iatrogenic prematurity (RCOG, 2014). Efforts to develop therapies for FGR are hampered by a lack of knowledge of the molecular mechanisms responsible for regulating blood flow through the placenta.

Clinically, assessment of placental haemodynamics using Doppler ultrasound (US) quantifies the ‘differences between the peak systolic and the end-diastolic velocity within blood vessels of interest in each cardiac cycle’ (Alfirevic *et al.*, 2015). This is given as the pulsatility index (PI) or resistance index (RI), and reflects the downstream vascular resistance within the placenta. In normal pregnancy umbilical arterial Doppler indices suggest a high flow/low resistance circulation. In those affected by FGR, high umbilical artery resistance is commonly observed, with the degree of abnormality corresponding to the level of placental compromise. This may result in absent or reversed blood flow at the end of diastole, indicating a foetus at high risk of FGR and stillbirth (Alfirevic *et al.*, 2015; Jones *et al.*, 2015).

The regulation of placental vasomotor tone relies on the production of vasodilators and vasoconstrictors from FpECs, given the lack of autonomic innervation (Rodríguez and González, 2014). A trigger for the release of vasoactive mediators from the endothelium is the frictional force, shear stress. This is defined as the ‘force exerted by the blood flow on blood vessel walls’, and is dependent upon the inner diameter of the vessel, flow rate, blood viscosity and pulsatility (Rodríguez and González, 2014). During each cardiac cycle, the frictional force of blood flowing across the vessel wall results in shear stress on the ECs lining the vessel lumen (Sprague *et al.*, 2010). This mechanosensing is involved in multiple vascular functions within the placenta, such as angiogenesis and blood pressure control, through the regulation of vascular tone and remodelling (James *et al.*, 2012; Ando and Yamamoto, 2013). Flow-mediated vasodilatation has been demonstrated in a perfused placental cotyledon model, with incremental increases in flow resulting in decreasing vascular resistance (Jones *et al.*, 2015).

There is paucity in the literature of other studies investigating the mechanisms responsible for the sensing, and adaptive response to shear stress in the placenta. Köhler *et al.* first described ‘stretch-activated cation channels’ in human umbilical vein endothelial cells (HUVECs) and found that they were involved in Ca^2+^ influx (Köhler *et al.*, 1998; Brakemeier *et al.*, 2002). Although the channel identity remained unknown in their study, they found a doubling in the density of the channels in HUVECs from pregnancies affected by preeclampsia (Köhler *et al.*, 1998). Understanding the mechanisms through which shear stress is sensed and how FpECs respond will provide novel insights into the control of placental vascular tone and therefore blood flow.

In 2014, our group first reported that the mechanosensitive ion channel subunit, Piezo1, responds to shear stress in a variety of EC types, including HUVECs (Li *et al.*, 2014). These Piezo1 channels are composed of Piezo1 proteins assembled as a large trimer of ~0.9 million Daltons in the cell membrane (Coste *et al.*, 2010; Ge *et al.*, 2015; Guo and MacKinnon, 2017; Beech and Xiao, 2018; Saotome *et al.*, 2018). Cryo-electron microscopy has revealed the structure of Piezo1 to be propeller-like, with three highly flexible blades. A central cap encloses the central gated ~25-pS calcium ion (Ca^2+^)-permeable non-selective cation channel (Ge *et al.*, 2015; Guo and MacKinnon, 2017; Saotome *et al.*, 2018; Zhao *et al.*, 2018). By assembling as large trimers the blade components act as ‘force sensors’ within the vessel lumen, acting to regulate the ion-conducting pore (Ge *et al.*, 2015; Guo and MacKinnon, 2017).

In addition to mechanical activation, a high-throughput screen by Syeda *et al.* (2015) identified Yoda1 as a chemical capable of activating Piezo1. To further investigate the significance of Piezo1, we generated a mouse model with a disrupted endogenous Piezo1 gene. Inheritance of the homozygous *Piezo1* deletion was embryonically lethal in 49 pups at mid-gestation (embryonic Days 9.5–11.5). Growth restriction of the embryos was commonly observed (embryonic Day 10.5), alongside reduced yolk sac vascularisation (Li *et al.*, 2014). These findings were also apparent in an endothelial-specific Piezo1 knockout murine model. Ranade *et al.* (2014) also found that mice homozygous for Piezo1 disruption developed FGR before dying at mid-gestation. This raises the question of whether a common molecular pathway involving Piezo1 also features in the abnormal placental function seen in FGR.

In human disease, a gain-of-function mutation in the Piezo1 gene has been already found in patients with hereditary xerocytosis, indicating the importance of Piezo1 for erythrocyte stability (Zarychanski *et al.* 2012). Additionally, in embryonic development, an autosomal recessive Piezo1 loss of function mutation is a cause of non-immune hydrops and therefore foetal demise (Fotiou *et al.*, 2015; Lukacs *et al.*, 2015). The role of Piezo1 as a mechanosensor in eukaryotic cells is a fast-paced area of research, with recent findings in the gastrointestinal tract epithelium, cardiomyocytes, chondrocytes and renal endothelium (Alcaino *et al.*, 2017; Allison, 2017; Liang *et al.*, 2017; Servin-Vences *et al.*, 2017). One example is the newly discovered role of Piezo1 in pressure-induced pancreatitis (Romac *et al.*, 2018). The authors describe pressure-induced Piezo1 channel activation. This was also induced by Yoda1 application and inhibited by Piezo1 antagonism (Romac *et al.*, 2018). Despite these findings highlighting the functional importance and pathological relevance of Piezo1, to our knowledge there have been no studies investigating Piezo1 in the placenta.

Further work is required to determine the importance of mechanosensing ion channels in the control of placental vascular tone, and in particular the role of Piezo1. In this study, FpECs have been isolated and cultured from within normal human placentas, to provide an *in vitro* method for investigating the presence, and function of Piezo1. In addition, human placental arterial cells from the chorionic plate have been used to determine the expression of Piezo1 in freshly isolated cells.

## Materials and Methods

### Study protocol and ethical approval

Patients delivering by elective caesarean section at Leeds Teaching Hospitals Trust were consented pre-operatively and provided with written information, in accordance with the approval granted by the local ethics committee (Ref 13/YH/03/44). Inclusion criteria were patients delivering at term, with no foetal growth or maternal health concerns. Patients with a history of smoking, BMI > 30 kg m^−^^2^, age > 40 years, diabetes mellitus, multiple pregnancy and babies with congenital abnormalities, were excluded. Information was collected on a proforma that included ultrasound findings, baby weight and sex. Placental tissue was sampled from the villous surface of the placenta within 30 min of delivery and weighed after removal of the umbilical cord and membranes. A 2 × 2 cm macroscopically-normal sample was taken from the villous surface of each of four quadrants and pooled. This tissue was transported to the laboratory on ice, in Endothelial Cell Growth Medium-2 (EGM-2; Lonza, USA) and processed immediately.

### Primary EC isolation and culture

Isolation of FpECs was undertaken in a sterile manner, using a protocol adapted from van Beijnum *et al.* (2008). Placental tissue was minced and re-suspended in a dissociation solution of 0.1% Collagenase II w/v, 2.5 units ml^−1^ Dispase w/v, 1 μM CaCl_2_ and 1 μM MgCl_2_ in Hanks Buffer solution. This was incubated at 37°C for 45 min in a MACSMix Tube Rotator (Miltenyi Biotech, UK) to provide continuous agitation. Samples were passed through 100 μm and 40 μm cell strainers to remove undigested tissue. Cells were washed in magnetically-activated cell sorting (MACS) buffer consisting of Phosphate Buffered Saline (PBS), 2 mM EDTA, and 0.1% Bovine Serum Albumin (BSA) w/v. Washed pellets were suspended in red blood cell lysis buffer consisting of 17 mM Tris base and 140 mM NH_4_Cl in PBS. Cells were washed for a final time in MACS buffer before being incubated with dead cell removal paramagnetic microbeads (Miltenyi Biotec). After incubation, cells were passed through a column prepared with binding buffer (Miltenyi Biotec) in a magnetic field (MiniMACS Separator, Miltenyi Biotec). The eluate consisting of live cells was incubated with Fc receptor blocking reagent and CD31-conjugated paramagnetic microbeads under continuous agitation. After incubation, the solution was passed through a column prepared with MACS buffer. CD31 positive cells were retained in the column and CD31 negative cells passed through as eluate. The CD31 positive cells were eluted from the column and re-suspended in culture media. FpECs were cultured in EGM-1 growth medium supplemented with EGM-2 bullet kit (Lonza, CC-3162). Cells were maintained at 37°C in a humidified atmosphere containing 5% CO_2_ and 95% air. Cells were used from passage 2–5. Images were taken using IncuCyte ZOOM Kinetic Imaging System (Essen BioScience, UK) and analysed with ImageJ software (Schneider *et al.*, 2012).

### Immunohistochemistry

FpECs were fixed on glass coverslips with paraformaldehyde solution then permeabilised with 0.1% Triton X-100 w/v. To prevent non-specific antibody binding, cells were blocked with donkey serum (5% v/v). Cells were incubated with primary antibody (anti-human CD31 JC70A, 1:300, Dako, USA; anti-human vWF F8/86, 1:200, Dako, USA; anti-vimentin C20 sc-7557, 1:100, Santa Cruz Biotechnology Inc., USA; anti-α-SMA-Cy3 1A4, 1:100, Sigma, UK; anti-cytokeratin peptide, 1:200, Sigma, UK), washed, then incubated with the relevant species-specific secondary antibodies conjugated with appropriate fluorophores. DAPI was used to counterstain cell nuclei. Slides were imaged using an LSM 880 confocal microscope (Zeiss, Sweden). Image analysis was performed using ImageJ to quantify staining intensity.

### Quantitative RT-PCR

Quantitative RT–qPCR (QPCR) was used to assess mRNA expression of *PIEZO1*. Total RNA was extracted from confluent FpEC colonies using a standard Tri-reagent protocol, followed by DNase treatment (TURBO DNA-free, Invitrogen, UK). QPCR was carried using Roche Fast Start SYBR Green I on a Lightcycler2 with Lightcycler 3.5 software or using 480 SYBR Green I on a LightCycler 480 II with LightCycler 1.5.62 software (Roche, UK). DNA amplification was for 35 cycles with an initial 10 min activation step at 95°C followed by 10 s at 95°C, 6 s at 55°C and 14 s at 72°C. Sequences of PCR primers were: Piezo1_Fwd, AGA TCT CGC ACT CCA T, Piezo1_Rv, CTC CTT CTC ACG AGT CC and Beta Actin_Fwd, TCG AGC AAG AGA TGG C, Beta Actin_Rv, TGA AGG TAG TTT CGT TGG ATG. PCR cycle crossing-points (CP) were determined by fit-points methodology. Threshold cycle (Ct) values were normalised to the housekeeping gene, beta actin, to obtain ΔCt values. All quantitative PCR reactions were performed in duplicate and the data averaged to generate one value per experiment. The specificity of QPCR was verified by performing a control reaction without reverse transcriptase (-RT), by melt-curve analysis and confirming amplicon size of PCR product on 2% agarose gels containing SYBR Safe DNA gel stain (Invitrogen, UK).

## Functional assays

### Intracellular Ca^2+^ concentration

Changes in intracellular Ca^2+^ concentration, were measured using a FlexStation 3 (Molecular Devices, USA) bench-top fluorometer. Cells were incubated with Fura-2 loading solution, consisting of the ratiometric Ca^2+^ indicator dye, Fura-2 AM (1 mM), and pluronic acid (10% w/v) in Standard Bath Solution (130 mM NaCl, 5 mM KCl, 1.2 mM MgCl_2_, 1.5 mM CaCl_2_, 8 mM d-glucose and 10 mM HEPES, pH 7.4). Stock solutions were prepared containing drugs to be tested (30 ng ml^−1^ VEGF/0.1–20 μM Yoda1/10 μM ATP/30 μM gadolinium chloride hexahydrate (Gd^3+^)). Baseline fluorescence ratios were recorded before the addition of the compound solution to the cells after 60 s, with regular recordings for a total of 6 min.

### Shear stress

Shear stress was achieved by placing cells on an orbital shaker (153 RPM) for 48 h, in a method described by Dardik *et al.* (2005) and Li *et al.* (2014). This produces tangential shear stress, resulting in cell elongation and alignment. ImageJ software was used to analyse cell images taken on the Incucyte, where the OrientationJ plugin37 quantified cell orientation relative to the direction of shear stress (http://bigwww.epfl.ch/demo/orientation/). A Gaussian distribution curve was fitted to each arising histogram. The baseline-subtracted frequency maximum at the mode of the distribution was determined.

### Angiogenesis

Total of 20 000 FpECs/well were plated into a 96-well plate containing growth factor-reduced Matrigel® (10 mg ml^−1^; Corning, USA). After 8 h, images of the tube-like structures were captured on the Incucyte. Staurosporine (SSP; 1 μM) was used as a negative control for tube formation. This chemotherapeutic agent induces cell death via intrinsic apoptotic pathways (Belmokhtar *et al.*, 2001). *In vitro* tube formation was also assessed using an endothelial/fibroblast co-culture assay. Here, Normal Human Dermal Fibroblasts (NHDF, Lonza) were seeded at 6 000 cells per well in a 96 well plate (Greiner Bio-one, UK) and allowed to grow to a confluent monolayer. FpECs were seeded on top of the fibroblast monolayer and allowed to grow into tube-like structures. CD31 immunocytochemistry was then performed (as above) and number of tubes quantified using the Incucyte.

### Piezo1 depletion and chemical activation

Confluent FpECs were transfected with 20 nM siRNA using Lipofectamine 2000 in OptiMEM (Invitrogen, UK). The siRNA probe sequence was GCCUCGUGGUCUACAAGAUtt (Ambion, UK). Control siRNA (non-targeting, scrambled) was obtained from Dharmacon, UK (http://dharmacon.gelifesciences.com/sirna/on-targetplus-non-targeting-control-pool/). Cells were incubated with the transfection mix added to EGM-2 for 3.5 h, before removing and replacing with fresh EGM-2 media. Experiments were performed after 48 h. Piezo1 knockdown was confirmed by QPCR, using beta actin as a house keeping gene. Cell viability post transfection was confirmed using a LIVE/DEAD Cell Imaging Kit (ThermoFisher, UK), which recognises plasma membrane integrity. The synthetic compound, Yoda1 (0.01–20 μM), was used to chemically activate Piezo1 in FpECs. In addition to siRNA, the blocker of voltage-sensitive Ca^2+^ channels, Gd^3+^, was used to determine the effect of Piezo1 inhibition on the Ca^2+^ response to Yoda1.

### Freshly isolated placental artery ECs

Human placental arterial ECs were isolated from second order arteries from the chorionic plate within 30 min of delivery, in a method previously described and validated (Greenberg *et al.*, 2016; Rode *et al.*, 2017). In brief, the placental arteries were enzymatically digested in dissociation solution (126 mM NaCl, 6 mM KCl, 10 mM Glucose, 11 mM HEPES, 1.2 mM MgCl_2_, 0.05 mM CaCl_2_, with pH adjusted to 7.2) containing 1 mg ml^−1^ collagenase Type IA (Sigma-Aldrich, UK) for 14 min at 37°C before they were triturated gently to release the ECs onto a glass coverslip.

### Patch-clamp electrophysiology

Outside-out membrane patch recordings on freshly isolated human placental arterial ECs were made with an Axopatch-200A amplifier (Axon Instruments, Inc., USA) equipped with Digidata 1440 A and pCLAMP 10.6 software (Molecular Devices, USA) at room temperature. Heat-polished patch pipettes with tip resistances between 3 and 5 MΩ were used. For the application of fluid flow, membrane patches were manoeuvred to the exit of a capillary tube with tip diameter of 350 μm, out of which ionic (bath) solution flowed at 20 μl s^−1^. The external solution for recording consisted of 135 mM NaCl, 4 mM KCl, 2 mM CaCl_2_, 1 mM MgCl_2_, 10 mM glucose and 10 mM HEPES (pH 7.4) while the pipette solution was composed of 145 mM KCl, 1 mM MgCl_2_, 0.5 mM EGTA and 10 mM HEPES (pH 7.2).

## Statistical analyses

OriginPro® 8.6 (OriginLab, USA) software was used for data analyses and presentation. Data are expressed as mean ± standard error of the mean. Statistical comparisons were made using the Student’s *T*-test and one-way ANOVA. The number of experiments on independent patient samples is indicated by ‘n’. The number of replicates per independent experiment is denoted by ‘N’. Data were considered significant when *P* < 0.05.

## Results

### Characteristics of human fetoplacental endothelial cells (FpECs)

**Figure 1:**
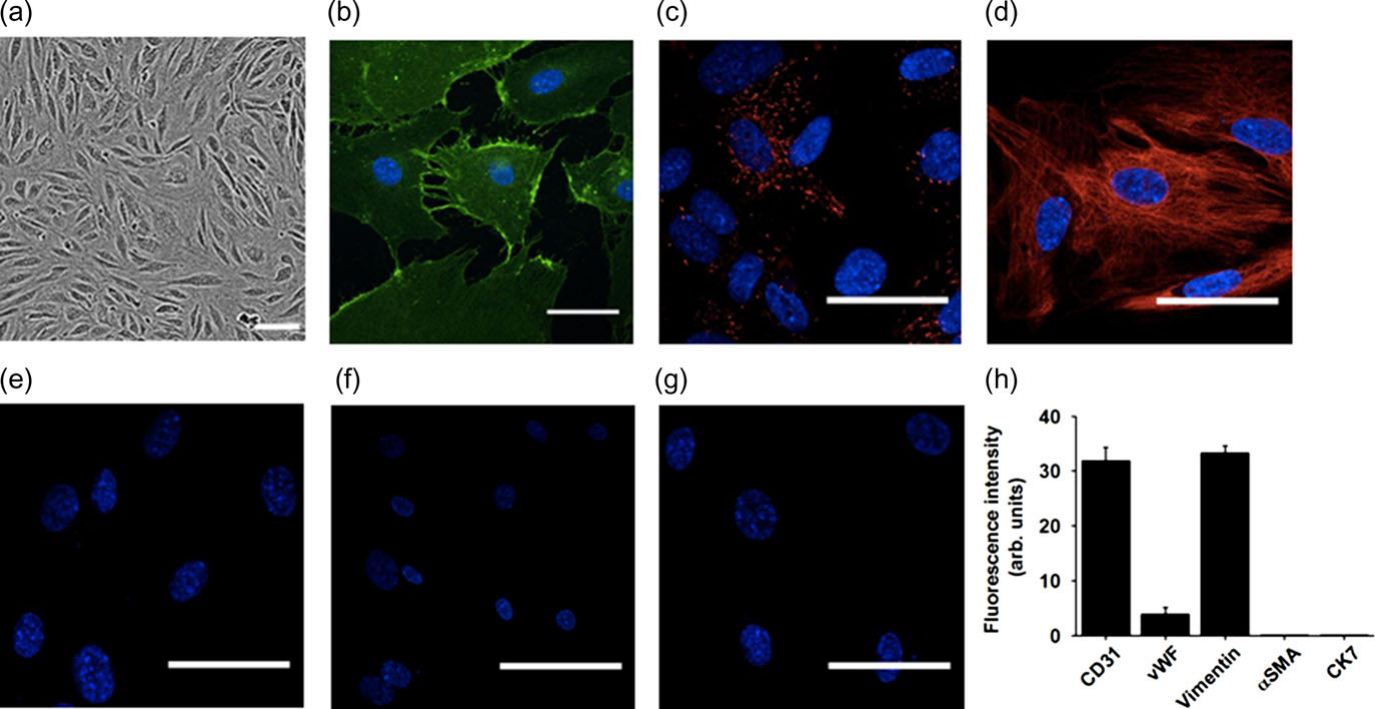
Protein and structural markers of fetoplacental endothelial cells (FpECs). **a**, phase contrast image of FpECs (scale bar, 100 μm). **b**–**e**, FpECs stained with: **b**, CD31 (green); **c**, von Willebrand factor (red); **d**, Vimentin (red); **e**, secondary antibody only (red, not visible). Scale bars, 50 μm. **f**, negative staining for the trophoblast marker cytokeratin 7 (CK7) (red, not visible). **g**, negative staining for the fibroblast marker α-SM actin (red, not visible). **b**–**g**, Cell nuclei labelled with DAPI (blue). **h**, quantification of FpEC staining intensity.

Immunohistochemistry was used to characterise the cultured FpECs (Fig. 1). They showed immunofluorescence for the endothelial marker CD31/PECAM-1 with its characteristic subcellular localisation to plasma membrane and adherens junctions (Fig. 1b). They expressed von Willebrand factor (vWF) in the expected punctate structures expected of Weibel-Palade Bodies, which carry vWF factor and are specific to ECs (Fig. 1c). Furthermore, they showed the characteristic expression of filamentous vimentin (Fig. 1d). Similar fluorescence was absent from controls in which primary antibodies for the endothelial proteins were excluded (Fig. 1e). Trophoblast (CK7) and fibroblast/smooth muscle (αSMA) marker proteins were not detected (Fig. 1f–h). These data suggest that FpECs share fundamental and unique proteins and structures of the general EC classification.

**Figure 2:**
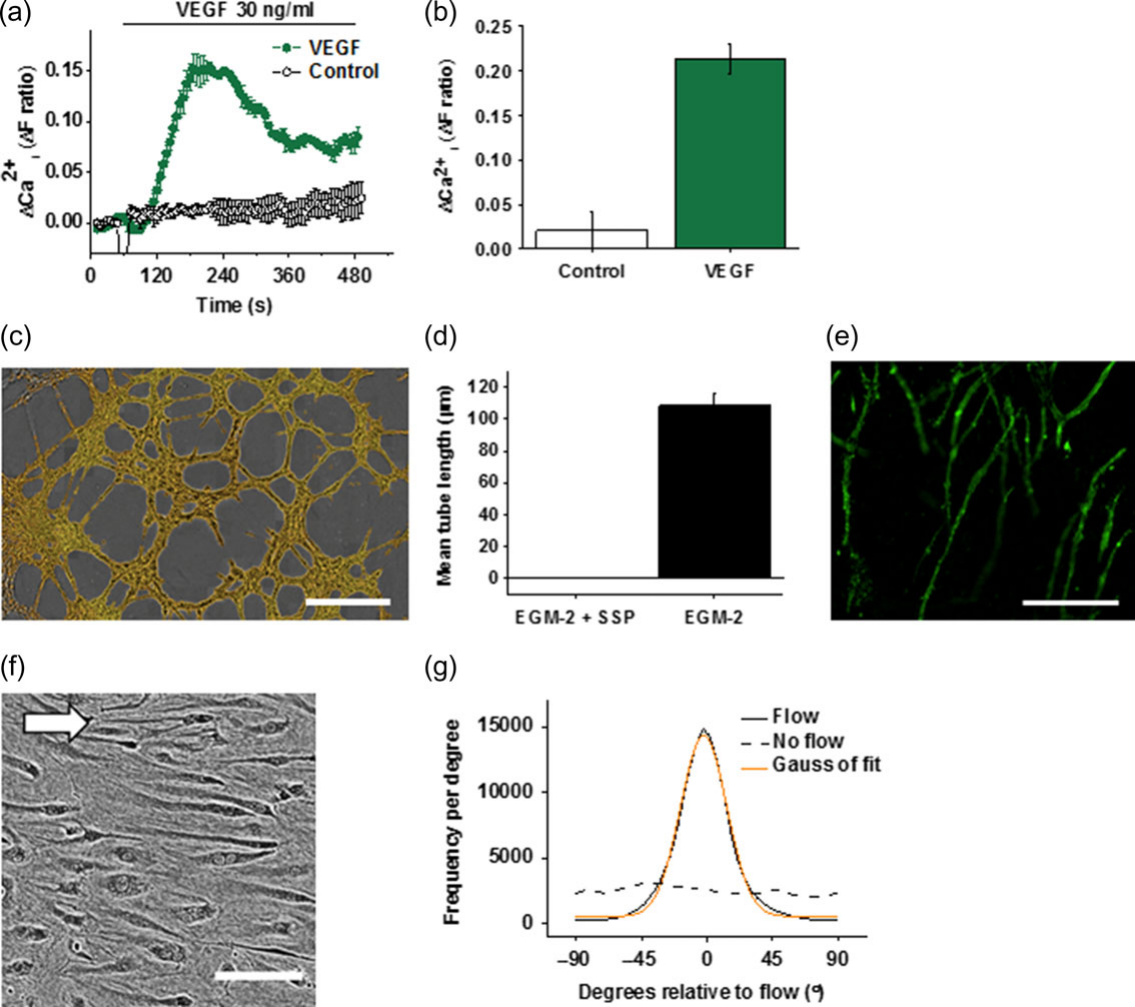
Angiogenic and shear stress-sensing properties of fetoplacental endothelial cells (FpECs). **a**, response of FpECs to vascular endothelial growth factor (VEGF; 30 ng ml^−1^) demonstrated by the intracellular Ca^2+^ elevation in multiple wells of a 96-well plate on a fluorescence plate-reader. **b**, VEGF response quantified against control (water): *P* < 0.05 (*n* = 3/*N* = 5). **c**, example image of tube formation on Matrigel (scale bar, 100 μm). **d**, as for **c** but quantified tube length of FpECs in endothelial cells growth medium 2 (EGM-2) in the absence or presence of 1 μM staurosporine (SSP) (*n* = 3/*N* = 5). **e**, example image of FpEC tube formation in co-culture with fibroblasts. The FpECs were labelled with anti-CD31 antibody (green). Fibroblasts are not visible in the image (scale bar, 300 μm). **f**, FpECs after exposure to 48 h of shear stress caused by an orbital shaker at 153 rpm, showing alignment in the direction of flow (arrow depicts direction of flow, scale bar 100 μm). **g**, orientation analysis for images of the type shown in **f**: solid line: cells exposed to flow, dashed line: cells in static culture, orange line: Gaussian fit curve.

Functional experiments showed that FpECs exhibited intracellular Ca^2+^ elevation in response to vascular endothelial growth factor (VEGF) (Fig. 2a and b) and that culturing of the cells on Matrigel (a solubilised basement membrane preparation) or a bed of human fibroblasts led to the formation of tube-like structures (Fig. 2c–e). Another common characteristic of ECs is their alignment in the direction of shear stress. Application of shear stress led to striking alignment of the FpECs to the direction of fluid flow (Fig. 2f and g). The data suggest that FpECs have mechanisms for angiogenesis and for the sensing of shear stress and alignment to the orientation of that shear stress.

### Piezo1 channels are a major Ca^2+^ entry mechanism in FpECs

**Figure 3:**
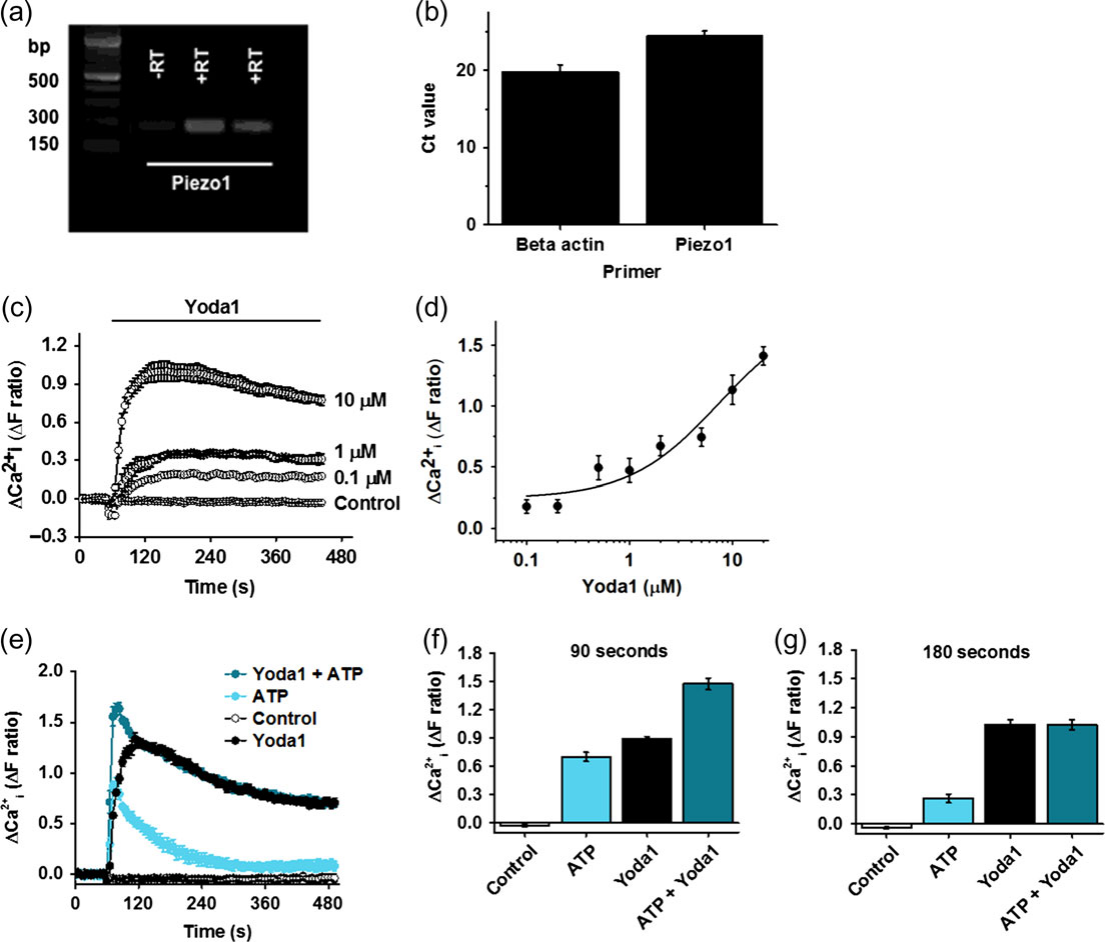
Expression of *PIEZO1* in fetoplacental endothelial cells (FpECs) and Ca^2+^-entry in response to Yoda1. **a**, gel electrophoresis showing *PIEZO1* mRNA following QPCR (182 bp). **b**, mean ± s.e. mean Ct values on QPCR for *PIEZO1* mRNA expression versus beta actin. **c**, change (Δ) in intracellular Ca^2+^ in response to increasing concentrations of Yoda1 (μM) compared with vehicle control. **d**, mean ± s.e. mean responses to Yoda1 fitted with the Hill equation, suggesting an approximate EC_50_ of 5.36 μM (*n* = 5/*N* = 3). **e**, intracellular Ca^2+^ responses after stimulating FpECs with Yoda1 (5 μM), ATP (10 μM) or a combination of Yoda1 (5 μM) and ATP (10 μM), versus DMSO control (*n* = 3/*N* = 3). **f**, mean ± s.e. mean data for the type of experiment shown in **e**, with values taken at 90 s (drug application at 60 s), showing a significant difference (*P* < 0.05) for ATP or Yoda1 alone versus both agonists. **g**, as for f, with data taken at 180 s, demonstrating a significant difference between ATP and ATP plus Yoda1 (*P* < 0.05), but a loss of significance for Yoda1 versus combination treatment.

**Figure 4:**
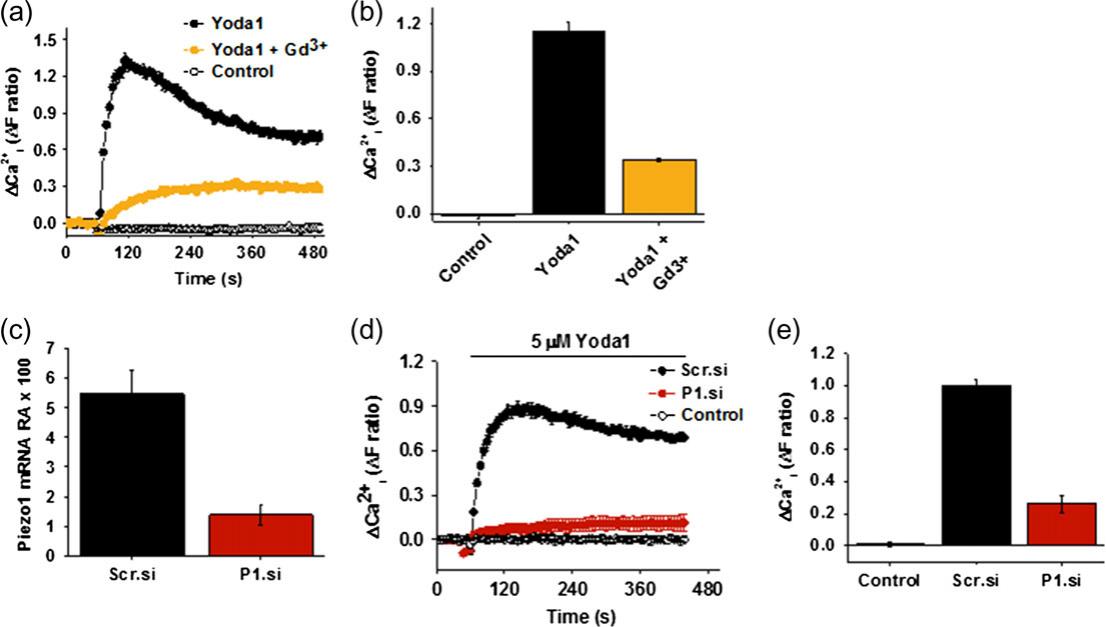
Piezo1-dependent Ca^2+^-entry in fetoplacental endothelial cells (FpECs). **a**, reduced intracellular Ca^2+^ response to Yoda1 after application of the calcium channel blocker Gd^3+^ (*n* = 3/*N* = 3). **b**, mean ± s.e. mean data for the type of experiment shown **a**, showing a significant difference in response between Yoda1 versus Yoda1 + Gd^3+^ application (*P* < 0.05). **c**, relative abundance of Piezo1 mRNA (normalised to beta actin) after treatment of FpECs with 20 nM scrambled siRNA (Scr.si) or 20 nM Piezo1 siRNA (P1.si) showing a significant reduction (*P* < 0.05). **d**, reduced intracellular Ca^2+^ response to Yoda1 after transfection with 20 nM P1.si or 20 nM Scr.si (*n* = 3/*N* = 5). **e**, mean ± s.e. m data for the type of experiment shown in **d**, showing a significant difference in response to Yoda1 between the Scr.si versus P1.si-treated cells (*P* < 0.05).

To establish whether the *PIEZO1* gene is expressed in FpECs, QPCR was performed on mRNA isolated from the FpECs. *PIEZO1* expression was readily detected (*n* = 10) (Fig. 3a, b). To investigate whether the mRNA leads to functional Ca^2+^-entry, the synthetic Piezo1 channel agonist Yoda1 was applied during measurement of the intracellular Ca^2+^ concentration (Fig. 3c). Yoda1 caused concentration-dependent increase in Ca^2+^ with 50% effect (EC_50_) occurring at about 5.4 μM (Fig. 3c and d). Because of aqueous solubility limitations of Yoda1 it was not possible to precisely determine the concentration of Yoda1 required for maximum effect. Therefore, the EC_50_ is an approximate estimate. The amplitude of the Yoda1 (5 μM) response was compared to the ATP (10 μM) response and found to be larger, particularly after 3 min, and more sustained (Fig. 3e–g). When ATP and Yoda1 were used in combination, a larger Ca^2+^ response was initially observed but this additive effect was quickly lost such that the combined response became indistinguishable from the response to Yoda1 alone (Fig. 3e–g). Blockade of Piezo1 using Gd^3+^, strongly suppressed the Ca^2+^ response to Yoda1 (Fig. 4a and b). Following these results, Piezo1 was depleted in the FpECs using siRNA. Knock down of Piezo1 using the siRNA was confirmed on QPCR (Fig. 4c). The response to Yoda1 was again strongly inhibited, suggesting that the Yoda1 effect does indeed depend on Piezo1 (Fig. 4d and e). These data suggest that Piezo1 channels are a major Ca^2+^ entry mechanism in FpECs.

### Piezo1 is required for FpEC alignment to shear stress

**Figure 5:**
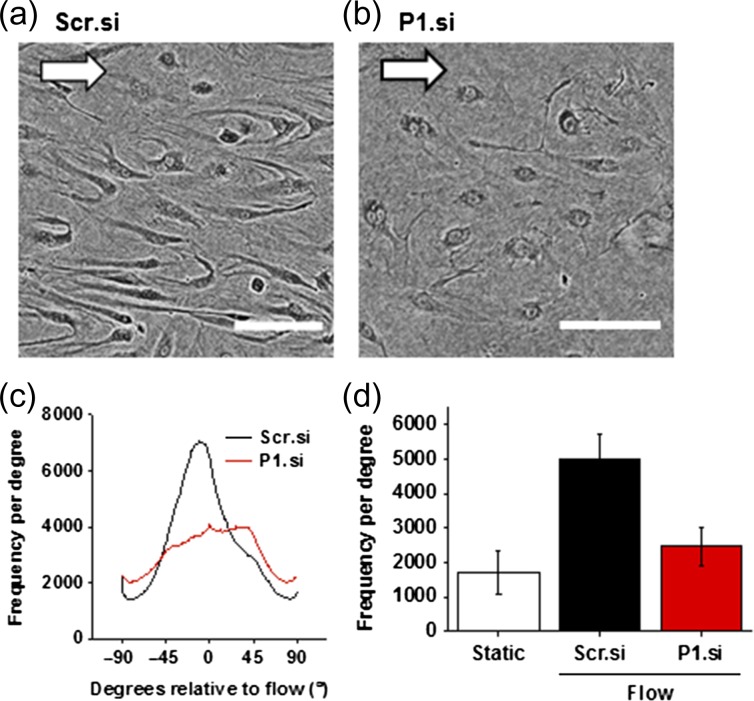
The response to shear stress in fetoplacental endothelial cells (FpECs) is Piezo1 dependent. **a**, FpECs transfected with scrambled siRNA after exposure to 48 h of shear stress caused by an orbital shaker at 153 rpm, showing alignment in the direction of flow (arrow depicts direction of flow, scale 100 μm). **b**, lack of alignment in FpECs exposed to shear stress after Piezo1 depletion with siRNA. **c**, orientation analysis for images of the type shown in **a** and **b**, showing the frequency (number of angles) at the mode (*n* = 3/*N* = 2). **d**, quantification of orientation analysis showing significantly reduced alignment after P1.si transfection (*P* < 0.05): mean height (SEM) after Scr.si 5674.611 (760.763), P1.si 2185.42 (548.786), static 1728.812 (632.188) (*n* = 3/*N* = 2).

Repeating the shear stress experiment in FpECs transfected with Piezo1 siRNA showed that FpECs no longer aligned in the direction of flow after Piezo1 depletion (Fig. 5a–c). This loss of alignment was significant between the control scrambled siRNA-transfected FpECs and the FpECs transfected with Piezo1 siRNA (Fig. 5d).

### Piezo1 is not required for FpEC viability or substrate attachment

**Figure 6:**
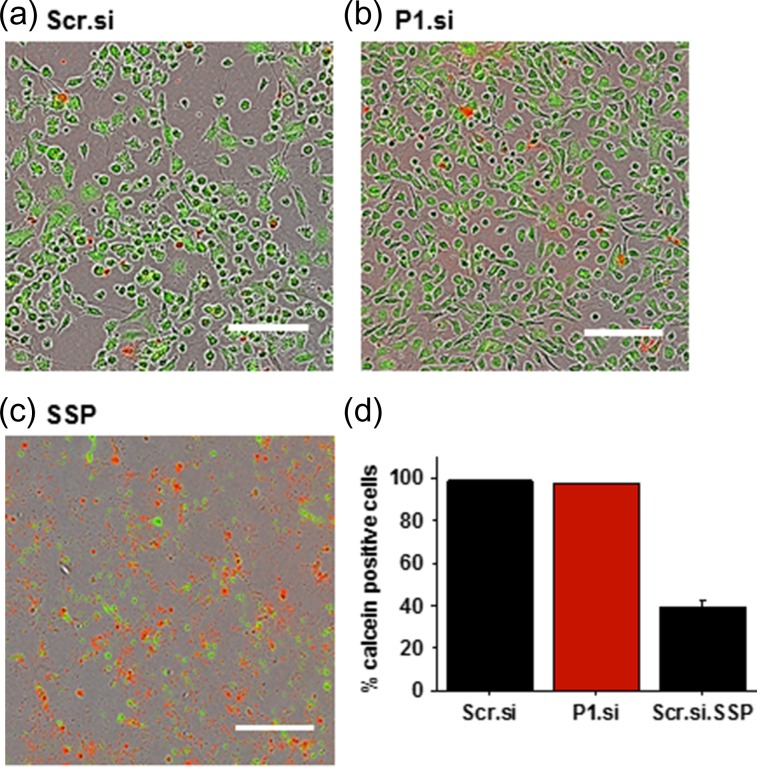
Piezo1 is not a requirement for cell viability or attachment to the substrate. Viability assay showing live cells of similar confluency in **a**, 48 h post transfection with Scr.si, **b**, 48 h after P1.si transfection, **c**, dead cell control post treatment with 1 μM staurosporine (SSP) (scale 300 μm). **d**, quantification showing no difference in viability between scrambled siRNA and P1.si transfected cells (p = non-significant). Treatment with SSP results in cell apoptosis and loss of cell attachment, verifying the viability assay.

An imaging assay that recognises plasma membrane integrity was used to confirm viability in FpECs 48 h after transfection with Piezo1 siRNA (Fig. 6a and b). Piezo1 depletion had no effect on the percentage of cells which were viable or attached to the substrate. Application of staurosporine (SSP) to cause apoptosis was a positive control and led to significant decrease in FpEC viability (Fig. 6c and d). These data suggest that Piezo1 is unimportant for the viability or substrate attachment of FpECs.

### Endothelium freshly isolated from human placental artery shows dominant shear stress-activated Piezo1 channels

**Figure 7:**
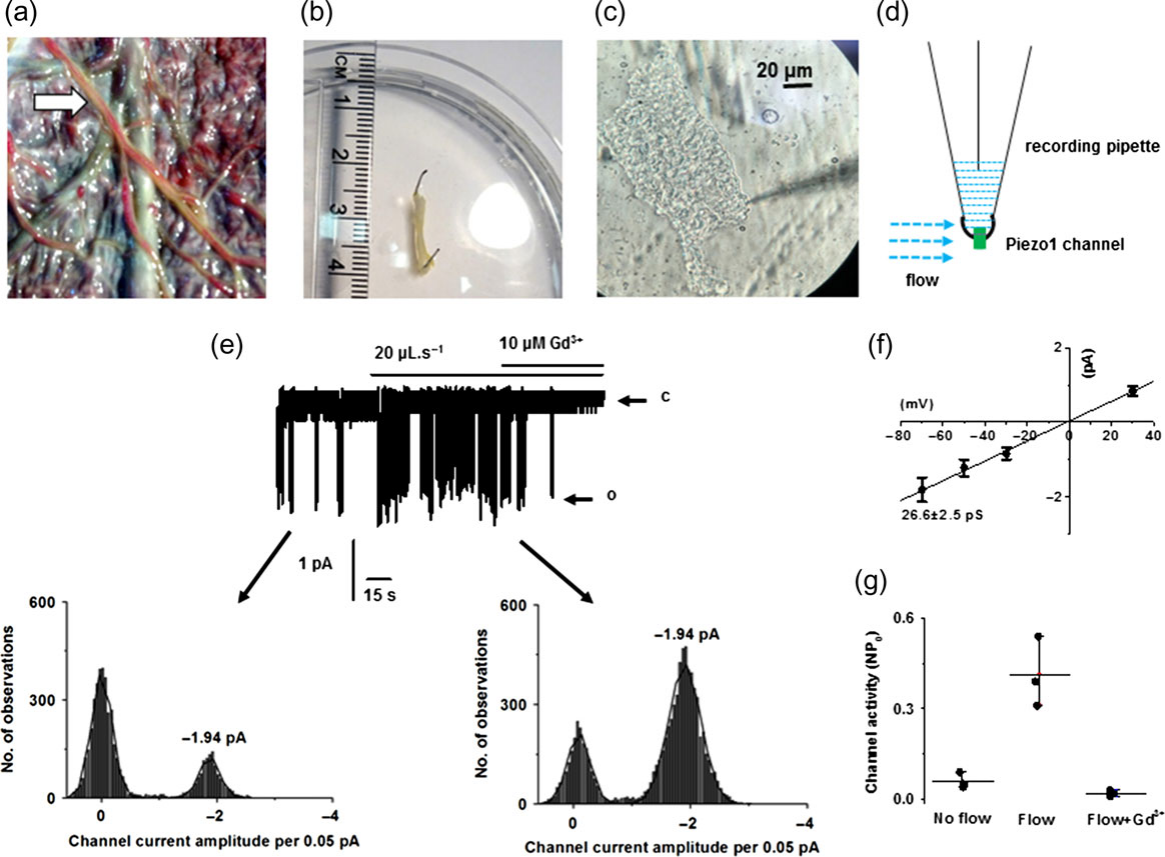
Piezo1 channels are membrane-delimited sensors of shear stress in freshly-isolated endothelium of human placental artery. **a**, placental chorionic plate. Arrow: artery to be isolated. **b**, placental artery isolated (diameter 760 μm), cleaned and placed in physiological solution. **c**, morphological appearance of freshly isolated placental arterial endothelial cell. **d**, drawing illustrating the method of ionic current recording from outside-out patches excised from freshly isolated endothelium of human placental artery. **e**, example recording at −70 mV. The patch was placed at the outlet of a capillary from which flowed ionic solution at 20 μl s^−1^. The letter c denotes the closed state of the channel and the letter o marks the open state. Subpanels show the current amplitude histograms for the segments of trace indicated by arrows. **f**, mean unitary current amplitudes for channels activated by flow as shown in **e** (*n* = 6/*N* = 3). **g**, raw data and mean data for channel activity (NP_o_: channel number × probability of opening) of the type shown in **e** (*n* = 3/*N* = 3). Flow *P* = 0.007, flow + Gd^3+^*P* = 0.004.

We considered that Piezo1 channel characteristics might be different in cells undergoing culture as compared to native endothelium. We therefore used freshly isolated placental arterial ECs for cell-detached outside-out membrane patch recording (Fig. 7a–d). This allowed the identification of single channel currents and investigation of channel unitary conductance (Fig. 7e–g). Constitutive channel activity was observed which could be further enhanced by fluid flow and inhibited by Gd^3+^ (Fig. 7e–g). Measurement of unitary currents using amplitude histograms showed that the unitary current of the spontaneous channels was the same as that of the flow-activated channels (Fig. 7e, lower graphs). Construction of a current–voltage relationship (IV) showed that the channel conductance was voltage-independent with amplitude of 26.6 pS (Fig. 7f). Statistical analysis further confirmed the channel sensitivity to Gd^3+^ (Fig. 7g). No other unitary current amplitudes were observed in response to fluid flow. These data suggest that Piezo1 channels are the dominant channel type activated by fluid flow in endothelium of human placental artery.

## Discussion

We report that primary FpECs can be isolated from term placentas by tissue digestion and CD31 microbeads, and can form functional colonies, as assessed by morphological, biophysical and biochemical criteria. The decision to investigate ECs from within the placental cotyledon rather than macrovascular HUVECs stems from the phenotypic and epigenetic differences between these cells, and those from the conduit cord vessels. This includes higher expression of vascular mediators in microvascular cells and differential gene expression (Lang *et al.*, 2001, 2003; Su, 2015). Importantly, sampling tissue from within the placental cotyledon includes ECs from the gas-exchanging terminal villi (Kingdom *et al.*, 2000).

Establishing this *in vitro* system for studying the fetoplacental endothelium, provides a novel insight into mechanosensing and the presence of the Piezo1 channel in the human placenta. Fresh endothelial cells were also isolated. These were obtained through vessel dissection from the chorionic plate arteries and are therefore from larger capacity vessels than the cultured FpECs. Patch-clamp electrophysiology experiments on these cells enabled us to obtain the unitary conductance and thus Piezo1 signature of 25 ~pS. Our data therefore demonstrate the consistent finding of Piezo1 channel activity in both cell types and show that these cells respond to mechanical force. When Piezo1 is depleted in FpECs, the response to shear stress and chemical stimulation of Piezo1 is significantly diminished. This suggests the importance of Piezo1 channels in shear stress sensing and the associated Ca^2+^ entry of FpECs.

It remains to be determined how flow activates Piezo1 channels but our data (Fig. 7) support earlier findings from native murine endothelium (Rode *et al.*, 2017) in suggesting that these channels are activated by flow in a membrane-delimited manner (i.e. in excised membrane patches). The effect of flow might be directly on the channels or via a closely-associated mechanism. Studies of overexpressed or reconstituted Piezo1 channels have provided compelling evidence that these channels directly sense membrane tension (Lewis and Grandl, 2015; Cox *et al.*, 2016; Syeda *et al.*, 2016). If this is the only mechanism for activation, flow might act by modulating membrane tension, via mechanisms which are currently unknown. Once the channels are activated, Ca^2+^ entry occurs which acts in part by activating calpain to enable EC alignment to flow (Li *et al.*, 2014).

Previous work by our group used titanium dioxide trapping coupled with mass spectrometry to identify proteins affected by Piezo1 in HUVECs, cultured in both static and shear stress conditions (Li *et al.*, 2014). After Piezo1 depletion in these cells, we found a reduction in endothelial nitric oxide synthase (eNOS) function, suggesting that Piezo1 had a regulatory role in vasodilation through nitric oxide (NO). Knock down of Piezo1 also resulted in the reduction of VEGF-evoked phosphorylation of eNOS at serine 1177 – known to enhance eNOS activity (Li *et al.*, 2014). Nitric oxide is a well known, potent vasodilator within the placental vascular tree (Boeldt *et al.*, 2011). As such, the downstream response of FpECs to shear stress may include activation of eNOS and subsequent NO production. This has been demonstrated in a placental perfusion model where changing haemodynamic factors (increasing viscosity or flow rate) stimulated NO release (Wieczorek *et al.*, 1995). Increased eNOS expression was also found in ovine FpECs after exposure to shear stress (Li *et al.*, 2004).

Where FGR is caused by placental dysfunction, the anatomy of placental vascular tree is altered, alongside endothelial dysfunction and a hypoxic environment (Kingdom *et al.*, 2000). As such, subsequent increases in transmural pressure in small vessels will heighten placental vascular tone (Krause *et al.*, 2013). This high vascular resistance generates greater shear stress forces (Rodríguez and González, 2014), Indeed, vessels and chorionic plate arterial ECs from FGR-affected placentas show reduced flow-mediated vasodilatation, and increased NO production (Krause *et al.*, 2013; Jones *et al.*, 2015). Correspondingly, Myatt *et al.* (1997) demonstrated increased eNOS expression in placental endothelium from FGR-affected placentas. We propose that Piezo1 may have a role in mechanosensing in fetoplacental endothelial cells, which could be upregulated in a high shear stress environment, leading to activation of downstream pathways such as NO.

In addition to NO, it remains possible that other extracellular or intracellular proteins may interact with and regulate Piezo1 channels. Our data show an early additive response to the application of both ATP and Yoda1. Indeed, shear stress is known to stimulate the release of ATP from the endothelium. Wang *et al.* (2016) showed that Piezo1 is required for shear stress-induced ATP release in human umbilical artery endothelial cells. They suggest that this Piezo1-dependent ATP response is mediated by pannexin channels. Downstream signalling here also resulted in eNOS activation, along with AKT phosphorylation (Wang *et al.*, 2016). Further experimental work is therefore required to determine the potential commonality between Piezo1 channel activity and ATP activation in the fetoplacental endothelium.

The role of Piezo1 channels may also differ between vascular beds. For example, we previously found Piezo1 to be an important mechanosensor in the murine mesenteric artery, enabling the redistribution of blood flow away from the gut in response to exercise (Rode *et al.*, 2017). This mechanism was Piezo1 dependent, causing vasoconstriction generated through opposition of endothelium-derived hyperpolarisation factor (EDHF), a well-known vasodilator (Rode *et al.*, 2017). This suggests that Piezo1 channels are widely found in the endothelium and have multiple important roles in vascular biology. It also suggests that Piezo1 may have different roles under certain circumstances, specific to the vascular bed. Future studies will therefore shed light on the mechanisms through which Piezo1 acts in the placenta.

To the best of our knowledge, this is the first publication suggesting a specific cation channel that may be central to mechanotransduction in human placental endothelium. Extending this research to pathological FpECs isolated from placentas affected by FGR may help to better understand the underlying dysfunction and its impact on the foetus. Our ability to manipulate Piezo1 chemically using Yoda1, in addition to shear stress, has the potential to provide a new therapeutic target for the treatment of placental vascular disease.
